# Genome-wide 5-hydroxymethylcytosine modification pattern is a novel epigenetic feature of globozoospermia

**DOI:** 10.18632/oncotarget.3163

**Published:** 2015-02-02

**Authors:** Xiu-Xia Wang, Bao-Fa Sun, Jiao Jiao, Ze-Chen Chong, Yu-Shen Chen, Xiao-Li Wang, Yue Zhao, Yi-Ming Zhou, Da Li

**Affiliations:** ^1^ Department of Obstetrics and Gynecology, Shengjing Hospital of China Medical University, Shenyang 110004, China; ^2^ Key Laboratory of Genomic and Precision Medicine, Beijing Institute of Genomics, Chinese Academy of Sciences, Beijing 101300, China; ^3^ Department of Cell Biology, Key Laboratory of Cell Biology, Ministry of Public Health, and Key Laboratory of Medical Cell Biology, Ministry of Education, China Medical University, Shenyang 110001, China; ^4^ Department of Medicine, Brigham and Women's Hospital, Harvard Institutes of Medicine, Harvard Medical School, Boston, MA 02115, USA

**Keywords:** 5-hydroxymethylcytosine, epigenetic, genomic imprinting, globozoospermia

## Abstract

Discovery of 5-hydroxymethylcytosine (5hmC) in mammalian genomes has excited the field of epigenetics, but information on the genome-wide distribution of 5hmC is limited. Globozoospermia is a rare but severe cause of male infertility. To date, the epigenetic mechanism, especially 5hmC profiles involved in globozoospermia progression, remains largely unknown. Here, utilizing the chemical labeling and biotin-enrichment approach followed by Illumina HiSeq sequencing, we showed that (i) 6664, 9029 and 6318 genes contain 5hmC in normal, abnormal, and globozoospermia sperm, respectively; (ii) some 5hmC-containing genes significantly involves in spermatogenesis, sperm motility and morphology, and gamete generation; (iii) 5hmC is exclusively localized in sperm intron; (iv) approximately 40% imprinted genes have 5hmC modification in sperm genomes, but globozoospermia sperm exhibiting a large portion of imprinted genes lose the 5hmC modification; (v) six imprinted genes showed different 5hmC patterns in abnormal sperm (GDAP1L1, GNAS, KCNK9, LIN28B, RB1, RTL1), and five imprinted genes showed different 5hmC patterns in globozoospermia sperm (KCNK9, LIN28B, RB1, SLC22A18, ZDBF2). These results suggested that differences in genome-wide 5hmC patterns may in part be responsible for the sperm phenotype. All of this may improve our understanding of the basic molecular mechanism underlying sperm biology and the etiology of male infertility.

## INTRODUCTION

Globozoospermia (also called round-headed sperm syndrome), characterized by 100% round-headed spermatozoa and lack of acrosome, is a rare but severe cause of male infertility [1]. Familial globozoospermia a suggests that globozoospermia most probably originates in spermatogenesis, specifically in acrosome formation and sperm head elongation, which is largely determined by genetic and epigenetic factors [1–4]. As is already known, sperm carry distinctive epigenetic modifications that are adjusted by reprogramming during the spermatogenesis and fertilization process [1, 2]. However, to date, little is known about epigenomics, especially the 5-hydroxymethylcytosine (5hmC) profiles in the pathophysiology of globozoospermia. 5hmC, a novel modified cytosine, is oxidized from 5-methylcytosine by the ten-eleven translocation family of proteins, and the discovery of 5hmC in mammalian genomes has excited the field of epigenetics [5]. In addition, 5hmC, as a unique and dynamic mark of cellular state, has been shown to be involved in diverse cellular processes, including transcriptional regulation, DNA methylation regulation, stem cell pluripotency and tumorigenesis [6]. Notably, recent studies have suggested that highly ordered alterations of 5hmC are potentially responsible for the differentiation of spermatogenic cells [7]. For this reason, the present study was undertaken to investigate comprehensive 5hmC profiling in normal, abnormal, and globozoospermia sperm by a chemical-labeling and biotin-enrichment approach followed by Illumina HiSeq sequencing, and to provide novel insight into the epigenetic-mediated dysfunction in the pathogenesis of globozoospermia.

## RESULTS

### Isolation and identification of normal, abnormal, and globozoospermia sperm

**Figure 1:**
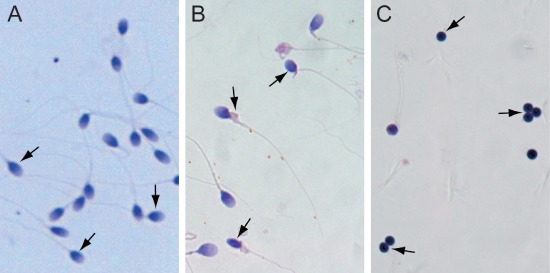
Isolation of normal sperm, abnormal sperm, and globozoospermia sperm **(A)** Normal sperm. **(B)** Abnormal sperm. **(C)** Globozoospermia sperm.

All normal sperm clearly have an oval head with a long tail (Figure 1A). While various misshapen head or tail defects such as amorphous head, crooked and double tail were observed in abnormal sperm (Figure 1B). The globozoospermia sperm had round heads (Figure 1C). In all, these three sets of sperm represent typical sperm types with different fertility ability and were used for studying the impact of 5hmC on male infertility.

### 5hmC enrichment and sequencing in normal, abnormal, and globozoospermia sperm

**Figure 2:**
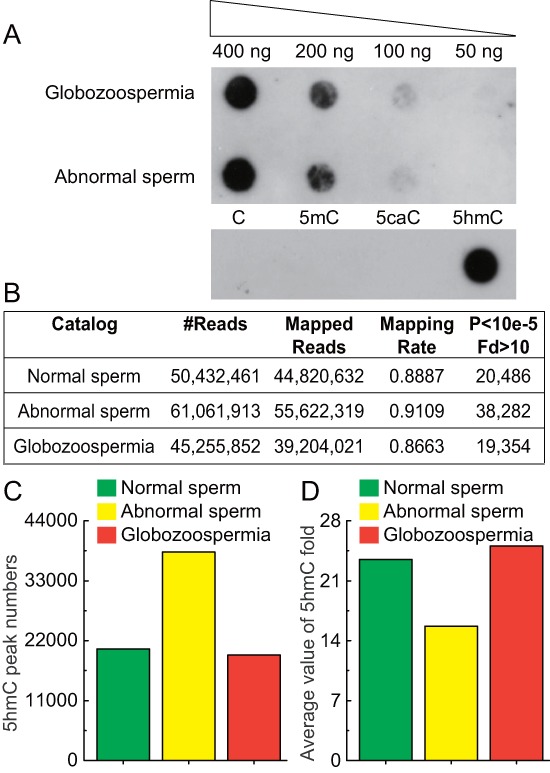
Sequencing results of 5hmC in normal, abnormal, and globozoospermia sperm genomes **(A)** Dot blot detection of 5hmC in sperm DNA with positive and negative control. 400 ng, 200 ng, 100 ng and 50 ng genomic DNA purified from abnormal and globozoospermia sperm were blotted on Hybond-N+ nylon-based membrane and immune-blotted using anti-5-hydroxymethylytosine antibody. Synthesized oligonucleotides with and without 5hmC modification were included as positive and negative controls. **(B)** Mapping results of 5hmC sequencing in normal, abnormal, and globozoospermia sperm genomes. Raw reads were aligned to human UCSC hg19 and peaks calling using MACS (*P* < 10^−5^, fold enrichment > 10). **(C)** 5hmC peak numbers in normal, abnormal, and globozoospermia sperms. 20486, 38282 and 19354 peaks were identified in normal, abnormal, and globozoospermia sperm, respectively. **(D)** Average value of 5hmC fold enrichment in normal, abnormal, and globozoospermia sperm.

To assess the general content of 5hmC, we first evaluated the existence of 5hmC in sperm genome by dot blot assay (Figure 2A). We detected a significant quantity of 5hmC in as little as 100 ng sperm genomic DNA. It is quite interesting to note that the amount of 5hmC is remarkably changed in different tissues in contrast to the stable patterns of 5-methylcytosine (5mC) [8].

To generate genome-wide maps of 5hmC in sperm genome, we used a well-established chemical-labeling and biotin-enrichment approach to enrich 5hmC-containing DNA fragments from normal, abnormal, and globozoospermia genomic DNA and subjected them to high throughput sequencing. Generally, we got 45 million to 60 million sequencing reads and mapped these to human genome with approximately 90% successful mapping rates (Figure 2B). We identified 5hmC enriched peaks using a model-based analysis of CHIP-seq software (MACS) (*P* < 10^−5^, fold enrichment > 10). In total, we identified 20486, 38282 and 19354 peaks in normal, abnormal, and globozoospermia sperm, respectively (Figures 2C and 2D, Supplementary Table S1).

### Genomic features of 5hmC in normal, abnormal, and globozoospermia sperm

**Figure 3:**
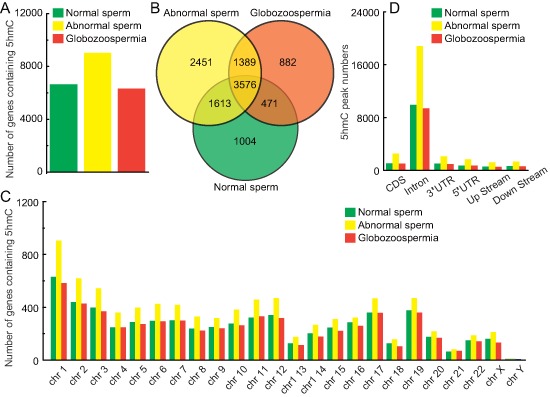
Genomic features of 5hmC in normal, abnormal, and globozoospermia sperm genomes **(A)** Number of genes containing 5hmC in normal, abnormal, and globozoospermia sperm genomes. **(B)** Overlap comparison of 5hmC-containing imprinted genes in normal, abnormal, and globozoospermia sperm genomes. **(C)** Number of genes containing 5hmC in each chromosome in normal, abnormal, and globozoospermia sperm genomes. **(D)** 5hmC peak numbers in CDS, intron, 3′UTR, 5′UTR, upstream (200 bp) and downstream (200 bp) in normal, abnormal, and globozoospermia sperm genomes.

We plotted those 5hmC peaks on Ref Seq annotated genes and identified 6664, 9029 and 6318 genes containing 5hmC in normal, abnormal, and globozoospermia sperm, respectively (Figure 3A), of which there was an especially strong overlap with 3576 genes in all these 5hmC gene pools (Figure 3B). The total and specific 5hmC-containing gene lists are shown in Supplementary Table S2. Furthermore, analysis of genome-wide 5hmC-containing genes shows that 5hmC are not distributed randomly on chromosomes, but exhibit a unique pattern on specific chromosomes (Figure 3C). With regards to the distribution region, it is striking that most of 5hmC peaks are located in introns (Figure 3D), whereas in ES cells 5hmC is preferentially present in the upstream of gene bodies and in the brain it is enriched in gene bodies [9, 10].

### GO analysis of 5hmC-containing genes in normal, abnormal, and globozoospermia sperm

**Figure 4:**
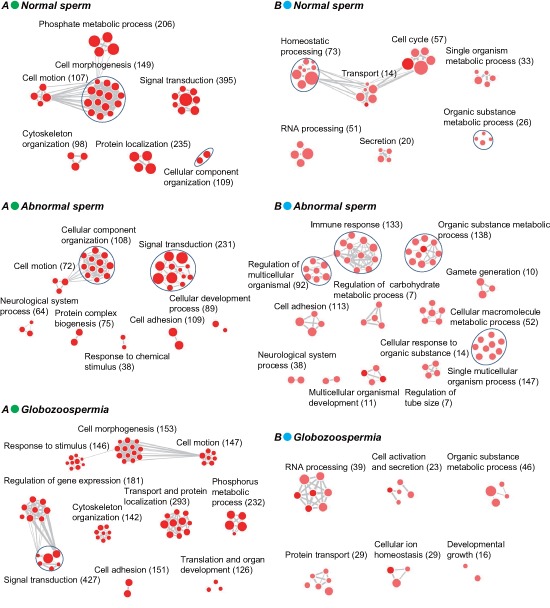
GO analysis of total and specific 5hmC-containing genes in normal, abnormal, and globozoospermia sperm genomes **(A)** GO analysis of total 5hmC-containing genes in normal, abnormal, and globozoospermia sperm genomes. **(B)** GO analysis of specific 5hmC-containing genes in normal, abnormal, and globozoospermia sperm genomes.

The preferential distribution of 5hmC in introns in sperm genome suggested it may have distinct roles in sperm maturation and function. As shown in Figure 4A and Supplementary Table S3, 5hmC-containing genes in all three genomes share cell motion and signal transduction pathways, indicating 5hmC has conserved but important roles in sperm motion and communication. Notably, cellular component organization is lost in globozoospermia but present in normal and abnormal sperm, whereas cell adhesion and response to (chemical) stimulus pathways is additionally involved in abnormal and globozoospermia without normal sperm.

To evaluate aberrant 5hmC modification in sperm dysregulation, we further performed GO analysis of specific 5hmC-containing genes in normal, abnormal, and globozoospermia sperm genome (Figure 4B, Supplementary Table S3). We found the organic substance metabolic process pathway is most significantly anomalous in normal, abnormal, and globozoospermia sperm. In particular, 10 gamete generation genes are implicated in abnormal sperm (Table 1), suggesting aberrant 5hmC modification of these genes may affect gamete generation, potentially leading to sterility of abnormal sperms.

**Table 1:** List of 5hmC containing genes associated with gamete generation in abnormal sperm

Gene symbol	Description
NOBOX	Homeobox protein NOBOX, Homeodomain-containing protein OG-2, Newborn ovary homeobox protein, Oocyte-specific homeobox protein
HEXB	Beta-hexosaminidase subunit beta, Beta-N-acetylhexosaminidase subunit beta, Hexosaminidase subunit B, Cervical cancer proto-oncogene 7 protein, N-acetyl-beta-glucosaminidase subunit beta
GDF9	Growth/differentiation factor 9 (GDF-9)
REC8	Meiotic recombination protein REC8 homolog (Cohesin Rec8p)
SOHLH2	Spermatogenesis- and oogenesis-specific basic helix-loop-helix-containing protein 2
FIGLA	Factor in the germline alpha, Class C basic helix-loop-helix protein 8, Folliculogenesis-specific basic helix-loop-helix protein, Transcription factor FIGa
ANG	Angiopoietin-4 (ANG-4) (Angiopoietin-3) (ANG-3)
ZGLP1	GATA-type zinc finger protein 1 (GATA-like protein 1) (GLP-1)
PAQR7	Membrane progestin receptor alpha (mPR alpha) (Progestin and adipoQ receptor family member 7) (Progestin and adipoQ receptor family member VII)
EREG	Proepiregulin

### 5hmC-containing genes overlap with imprinted genes among normal, abnormal, and globozoospermia sperm

**Figure 5:**
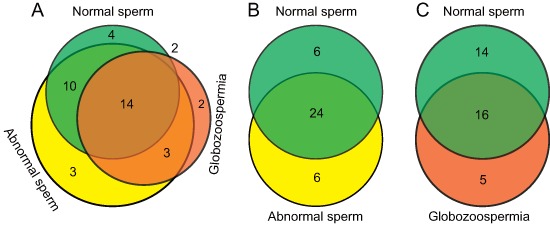
Overlap comparison of 5hmC-containing imprinted genes among normal, abnormal, and globozoospermia sperm by Venn diagrams **(A)** Overall comparison of 5hmC-containing imprinted genes among normal, abnormal, and globozoospermia sperm. **(B)** Overlap comparison of 5hmC-containing imprinted genes between normal and abnormal sperm. **(C)** Overlap comparison of 5hmC-containing imprinted genes between normal and globozoospermia sperm.

To evaluate 5hmC modification alteration in imprinted genes, we compared 5hmC-containing genes with 96 known imprint genes from the imprinted gene database (http://www.geneimprint.com/site/home), and visualized by area-proportional Venn diagrams using an online tool BioVenn. In total, approximately 40% of the imprinted genes (38 imprinted genes) are 5hmC-containing genes in normal, abnormal, and globozoospermia sperm genomes (Figure 5A). In detail, 30, 30 and 21 imprinted genes contained 5hmC in normal, abnormal, and globozoospermia sperm genomes, respectively (Table 2). The Venn diagram shows that normal, abnormal, and globozoospermia sperm share 14 imprinted genes (Figure 5A). Compared with normal sperm, 6 imprinted genes lost 5hmC modification, while another 6 imprinted genes gained 5hmC modification in abnormal sperm (Figure 5B). Interestingly, compared with normal sperm, a large portion (14 out of 30) of imprinted genes lost 5hmC modification and 5 imprinted genes gained 5hmC modification in globozoospermia patient (Figure 5C), suggesting that the loss of 5hmC in imprinted genes may be associated with globozoospermia.

**Table 2:** List of imprinted genes overlapped with 5hmC containing genes in sperm

Normal sperm	Abnormal sperm	Globozoospermia
AIM1	AIM1	ANO1
ANO1	ANO1	ATP10A
ATP10A	ATP10A	DDC
BLCAP	CDKN1C	DLGAP2
CDKN1C	DDC	GLIS3
DDC	DLGAP2	IGF2R
DGCR6L	GDAP1L1	INPP5F
DLGAP2	GLIS3	KCNK9
DNMT1	GNAS	KCNQ1
GLIS3	GPR1	LIN28B
GPR1	GRB10	MAGI2
GRB10	IGF2R	NLRP2
IGF2R	KCNK9	NTM
INPP5F	KCNQ1	OSBPL5
KCNQ1	LIN28B	PPP1R9A
MAGI2	MAGI2	RB1
MEST	MEST	SLC22A18
NLRP2	NLRP2	SLC22A3
NTM	NTM	TP73
OSBPL5	OSBPL5	ZDBF2
PLAGL1	PLAGL1	ZFAT
PPP1R9A	PPP1R9A	
SLC22A3	RB1	
SNRPN	RTL1	
TP73	SLC22A3	
UBE3A	SNRPN	
ZC3H12C	UBE3A	
ZFAT	ZC3H12C	
ZIM2	ZFAT	
ZNF597	ZIM2	

## DISCUSSION

Emerging evidence indicates that epigenetic mechanisms, especially the aberrant DNA methylation (5mC) of imprinted genes in sperm DNA, play an important part in abnormal sperm parameters and male infertility [11]. However, it is interesting to note that sperm methylation profiles have been recently described [12], but to date, no studies have examined the distribution and features of 5hmC in sperm genome, and few studies have linked 5hmC to male infertility. The current study generated the first landscape of 5hmC in normal, abnormal, and globozoospermia sperm genomes, and provided novel insights into 5hmC-related sperm physiology and pathology.

In this study, we identified 6664, 9029 and 6318 genes containing 5hmC in normal, abnormal, and globozoospermia sperm, respectively. Notably, 5hmC is exclusively localized in intron, a distinct characteristic difference from previous reports that 5hmC is preferentially enriched within exons and near transcriptional start sites in embryonic stem cells [10], and alteration of 5hmC modification occurred mainly at gene bodies, along with environmental changes [13] and neurons development [14]. In addition, the discrete distribution of 5hmC in the intron regions of sperm genome suggested that it may have specific roles in sperm maturation and function.

We also found that 5hmC-containing genes are involved in various functional pathways, some of which have important roles in sperm, such as spermatogenesis, sperm motility and morphology, and sperm cell maturation. For instance, (i) growth differentiation factor 9 (GDF9) is significantly associated with sperm quality traits, and is involved in the initiation or maintenance of spermatogenesis [15]; (ii) methylation patterns of the nuclear ribonucleoprotein polypeptide N (SNRPN) promoters are associated with changes in sperm motility and morphology, which could lead to male infertility [16]; (iii) membrane-associated guanylate kinase, WW and PDZ domain containing 2 (MAGI-2), which is known to localize at the tight junction of epithelial cells, plays an important part in sperm cell maturation [17]; (iv) FIGLA (factor in the germ line, alpha) encodes a germ cell-specific basic helix-loop-helix transcription factor, which has essential roles in the repression of sperm-associated genes during normal postnatal oogenesis [18].

It is well known that aberrant DNA methylation patterns, mainly in imprinted genes, have been associated with sperm dysfunction. Therefore, we further compared the 5hmC-containing genes among normal, abnormal, and globozoospermia sperm genomes, referring to the imprinted gene database. We found that approximately 40% (38 out of 96) imprinted genes had 5hmC modification in normal, abnormal, and globozoospermia sperm genomes. The globozoospermia sperm showed that a large portion (14 out of 30) of imprinted genes lose 5hmC modification, compared with normal sperm, suggesting that the loss of 5hmC in imprinted genes may have essential roles in globozoospermia progression. As shown in Table 2, six imprinted genes showed different 5hmC patterns between normal and abnormal sperm (GDAP1L1, GNAS, KCNK9, LIN28B, RB1, RTL1), and five imprinted genes showed different 5hmC patterns between normal and globozoospermia sperm (KCNK9, LIN28B, RB1, SLC22A18, ZDBF2). These data may help to identify several novel epigenetically regulated genes that are possibly involved in abnormal sperm and globozoospermia sperm, and these aberrant 5hmC-related genes may also be potential biomarkers of abnormal sperm parameters.

Taken together, our study provided a genome-wide distribution of 5hmC in normal, abnormal, and globozoospermia sperm, and understanding the epigenetic mechanisms may yield new insight into the sperm biology and etiology of male infertility.

## METHODS

### Ethical statements

The investigation was conducted in accordance with the ethical standards and according to the Helsinki Declaration of 1975, and was approved by the Institutional Review Board at China Medical University.

### Preparation of genomic DNA, 5hmC specific chemical labeling and affinity purification

Genomic DNA was prepared using a Wizard Genomic DNA Purification kit (Promega Cat.:A1120) by following the manufacturer's instructions. Equal amounts of genomic DNA were extracted from 1 × 10^5^ normal, abnormal, and globozoospermia sperm, respectively. Purified genomic DNA was sonicated into short fragments by Covaris DNA shearing with microTUBEs according to the manufacturer's instructions. Then 5hmC labeling reactions were performed in 75 μl solution containing 50 mm HEPES buffer (pH 7.9), 250 mm MgCl_2_, 100 μm UDP-6-N_3_-glucose and 80U β-glucosyltransferase. The reaction was incubated for 2 h at 37°C. After the reaction, DNA substrates were purified and buffer-exchanged in H_2_O via Bio-Rad-Spin columns according to the manufacturer's instructions. Click chemistry was performed with the addition of 150 μm biotin into the DNA solution and incubated for 2 h at 37°C. The DNA samples were then purified by Invitrogen Dynabeads MyOne™ Streptavidin C1 according to the manufacturer's instructions.

### Sequencing of 5hmC-enriched genomic DNA

5hmC-enriched genomic DNA libraries were generated following the Illumina protocol for ‘Preparing Samples for CHIP Sequencing of DNA’. Then, 20 ng of 5hmC-enriched DNA was used to initiate the protocol. DNA fragments were gel purified after the adapter ligation step. PCR-amplified DNA libraries were quantified on an Agilent 2100 Bio analyzer using a quantitative PCR. We performed 100 bp single end sequencing on Illumina Hiseq2000 to get a 5hmC-enriched DNA fragment sequence.

### 5hmC reads mapping and peaks calling

The deep sequencing reads were stripped of the adaptor sequences with FASTX tool kit (http://hannonlab.cshl.edu/fastx_toolkit/). Reads that were less than 25 nt in length or contained an ambiguous nucleotide were discarded. The remaining reads were aligned to human UCSC hg19 genome, with up to two mismatches allowed, by the BWA software [19]. All non-redundant uniquely mapped reads were used for peaks calling using MACS (*P* < 10^−5^) [20]. Association of 5hmC peaks with genomic features was performed by overlapping peak locations with known genomic features obtained from hg19 database. Location information of CDS, intron, 3′UTR, 5′UTR, upstream (200 bp), and downstream (200 bp) were downloaded from UCSC.

### Immuno-dot-blot assay

Genomic DNA was denatured in TE buffer for 10 min at 95°C and immediately chilled on ice for 5 min. Dot blot was performed on a Bio-Dot Apparatus (#170-6545, Bio-Rad); 50, 100, 200 and 400 ng of each DNA sample was spotted on the positively charged nylon membrane, respectively, then the membrane was baked for 2 h at 80°C until completely dry, followed by UV254 crosslink for 10 min to fix DNA on the membrane. The membrane was then blocked briefly with 5% non-fat milk for 1.5 h at room temperature. The primary rabbit anti-5-hydroxymethylytosine antibody (1:10000, #39769, Active Motif) was applied to the membrane and incubated at RT for 1 h or overnight at 4°C. After incubation with a peroxidase-conjugated anti-rabbit IgG secondary antibody, the signal was visualized by using ECL (Millipore). The dot-blot densities were analyzed with Image J software. The 5hmC-containing DNA was used as a positive control, and the normal C, 5mC, 5-carC-containing DNA were used as the negative controls to verify the specificity of 5hmC antibody.

### Accession number

All original data sets have been deposited in the Gene Expression Omnibus Database under the accession number GSE46135.

### Gene ontology analysis

Gene ontology analyses were performed on sets of unique RefSeq identifiers using DAVID bioinformatics resources 6.7 functional annotation tools [21]. GO Biological processes and Interpro database were used. Categories with *P* < 0.05 were considered statistically significant. The analysis results are visualized as an enrichment map by using Cytoscape software [22].

## SUPPLEMENTARY TABLES








