# Supplementary material for: Genome-wide 5-hydroxymethylcytosine modification pattern is a novel epigenetic feature of globozoospermia
Source: Oncotarget. 2015 Feb 2;6(9):6535–43. doi: 10.18632/oncotarget.3163 (PMC4466632; doi:10.18632/oncotarget.3163)
Supplement: Supplementary file 1 [file oncotarget-06-6535-s001.pdf]

SUPPLEMENTARY TABLES

Supplementary Table S1: 5hmC-enriched peaks in normal, abnormal, and globozoospermia sperm

Supplementary Table S2: Total and specific 5hmC-containing gene lists in normal, abnormal, and globozoospermia sperm

Supplementary Table S3: Functional analysis of 5hmC-containing genes in normal, abnormal, and globozoospermia sperm
